# Revisit to three-dimensional percolation theory: Accurate analysis for highly stretchable conductive composite materials

**DOI:** 10.1038/srep34632

**Published:** 2016-10-03

**Authors:** Sangwoo Kim, Seongdae Choi, Eunho Oh, Junghwan Byun, Hyunjong Kim, Byeongmoon Lee, Seunghwan Lee, Yongtaek Hong

**Affiliations:** 1 Department of Electrical and Computer Engineering, Inter-University Semiconductor Research Center (ISRC), Seoul National University, Seoul 08826, Republic of Korea

## Abstract

A percolation theory based on variation of conductive filler fraction has been widely used to explain the behavior of conductive composite materials under both small and large deformation conditions. However, it typically fails in properly analyzing the materials under the large deformation since the assumption may not be valid in such a case. Therefore, we proposed a new three-dimensional percolation theory by considering three key factors: nonlinear elasticity, precisely measured strain-dependent Poisson’s ratio, and strain-dependent percolation threshold. Digital image correlation (DIC) method was used to determine actual Poisson’s ratios at various strain levels, which were used to accurately estimate variation of conductive filler volume fraction under deformation. We also adopted strain-dependent percolation threshold caused by the filler re-location with deformation. When three key factors were considered, electrical performance change was accurately analyzed for composite materials with both isotropic and anisotropic mechanical properties.

There has been much interest in conductive composite materials because they can be used to fabricate highly stretchable electronic elements such as stretchable electrodes^1^,^2^,^3^ or stretchable sensors^4^,^5^,^6^. Particularly, recent development of advanced stretchable conductive composite materials for stretchable electrode application has shown high initial conductivity approaching conductivity of bulk metal, as well as much higher stretchability compared to metal thin films on stretchable substrates^3^,^7^,^8^,^9^,^10^,^11^,^12^. Such a great advancement in the material research has led to studies of conduction mechanism behind these excellent behaviors, where various analytical techniques including percolation theory, tunneling theory and inter particle distance (IPD) model^13^,^14^ were used as an attempt for precise prediction. However, since those previous models did not use deformation information in them, they intrinsically have limitation for accurate analysis. Therefore, by considering uni-axial stretching condition, an improved three-dimensional percolation theory was developed to explain the electrical behavior of the conductive composite materials depending on the applied tensile strain^10^. In this theory, volume fraction of conductive filler and percolation threshold, were determined by using volume variation from Poisson’s effect with elongation, and the IPD model, respectively. Several additional researches have shown various theoretical predictions of the conductive composite materials based on this three-dimensional percolation theory^12^,^15^,^16^,^17^.

As pointed out by Kim *et al.*^15^, however, the three-dimensional percolation theory also failed in precisely describing electrical behavior of the stretchable conductive composite materials, especially at high tensile strain region. This failure mainly arises from the improper usage of the linear and non-linear elasticity conditions. Although the three-dimensional percolation theory uses a large deformation assumption based on nonlinear elasticity condition, in previous researches, constant Poisson’s ratio, which is true under small deformation of linear elasticity condition, has been widely used in the analysis. It is well known that analysis of elastomers should be based on nonlinear elasticity with strain-dependent Poisson’s ratio^18^,^19^. Most of stretchable conductive composite materials that are commonly used in the field of stretchable electronics show nonlinear elasticity at large deformation and we should therefore consider proper conditions. If we use a constant Poisson’s ratio in calculation of the composite volume change under small to large deformation conditions, the theoretical composite volume shows impractical results, which are more apparent when the applied tensile strain is close to be 100% or larger. For example, the composite volume of a theoretical incompressible material with Poisson’s ratio of 0.5 decreases with the tensile strain and eventually vanishes at 200% tensile strain. Therefore, the three-dimensional percolation theory has been limitedly utilized for low deformation conditions and produced theoretical results that are not consistent with the experimental ones for the highly stretchable conductive composite materials. This inconsistency can be greater if the composite materials have anisotropic Poisson’s ratios. Therefore, we believe that a proper nonlinear elasticity condition with a corresponding strain tensor must be used to fully analyze the highly stretchable conductive composite materials from small to large strain conditions.

Another important factor for accurate analysis is an appropriate measurement of the Poisson’s ratio of the stretchable conductive composite materials. In the three-dimensional percolation theory, Poisson’s ratio noticeably affects variation amount of the volume fraction of conductive fillers in the composite materials depending on the applied tensile strain. Even a small error of Poisson’s ratio could result in a big distortion in the predicted conductive filler volume fraction. In spite of its significance, however, in previous researches on the stretchable conductive composite materials, they have not seriously dealt with measurement of the Poisson’s ratio and its effect on the analysis. There have no detailed experimental methods to measure the Poisson’s ratio in the previous reports. In fact, in order to guarantee a precise prediction from the three-dimensional percolation theory, Poisson’s ratios of the materials measured over the full strain ranges must be used. A reliable Poisson’s ratio can be obtained from an accurate measurement of the dimensional change of the composite materials under the tensile strain and adoption of the proper strain tensor well describing highly dynamic behavior of the materials. Under the large deformation condition, Hencky strain tensor is known to accurately explain behavior of an incompressible material without strain dependency, while Cauchy or Green tensor inaccurately describes the highly dynamic behavior of the highly deformable materials under large tensile strain ranges^20^,^21^,^22^.

Lastly, it is also important to use strain-dependent percolation threshold^15^ to properly explain the behavior of the stretchable conductive composite materials. Deformation along a certain direction could cause re-location of conductive fillers in the composite^9^. It is well-known that the percolation thresholds of two different composite systems are not same when the filler distribution is different even if the two composites have the same conductive fillers^14^. This means that percolation threshold could have a dynamic property due to conductive filler re-location from the deformation of the composite materials. We believe that this phenomenon also should be taken into account for the three-dimensional percolation theory under elongation and thus the corresponding percolation threshold should be regarded as a function of the applied strain. This phenomenon can be confirmed by using impedance spectroscopy for the composite materials under elongation condition.

In this paper, therefore, based on the factors mentioned above, we focused on the following three aspects to develop an improved three-dimensional percolation theory for accurate analysis of the electrical behavior of the stretchable conductive composite materials.

1. Strain-dependent Poisson’s ratio based on nonlinear elasticity
2. Accurate Poisson’s ratio using digital image correlation and Hencky strain tensor
3. Strain-dependent percolation threshold

The Hencky strain tensor was used in order to obtain reliable Poisson’s ratio from the measured dimensional change of the composite materials in two-dimensional directions perpendicular to the tensile strain direction under small to large deformation conditions. Digital Image Correlation (DIC) method^20^,^22^ was used to monitor dimensional change of the elongated composite materials.

## Results

### Validity of Strain-dependent Poisson’s Ratio in Large Deformation

Two important equations in the three-dimensional percolation theory used to calculate electrical conductivity (*σ*) of the conductive composite materials are described below for initial and elongated states.


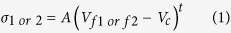



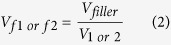


where index 1 or 2 describes initial or deformed state, respectively; *σ* is the electrical conductivity of conductive composite materials; *A* is proportionality constant related to the electrical conductivity of conductive fillers; *V*_*f*_ is volume fraction of conductive fillers; *V*_*filler*_ is volume of conductive fillers; *V*_*1*_ or *V*_*2*_ is volume of the conductive composites at initial and deformed state, respectively; *V*_*c*_ is percolation threshold which indicates the volume fraction where a rapid increase of conductivity is observed and has been considered a constant value in previous reports; and *t* is fitting exponent. The fitting exponent *t* is known as the factor related to the composite system’s dimensionality. As shown in the equations, determining the percolation threshold and the conductive filler volume fraction is important to derive the three-dimensional percolation theory. It is well known that, at the percolation threshold, almost all conductive composite materials show a sharp increase in their electrical conductivity even with an infinitesimal change in volume fraction of the conductive filler^23^. Since the volume fraction change is directly determined by variation of the total volume of the composite materials (*V*_*filler*_ is constant before and after deformation in almost all cases), accurate estimation of the total volume change is also important.

**Figure 1:**
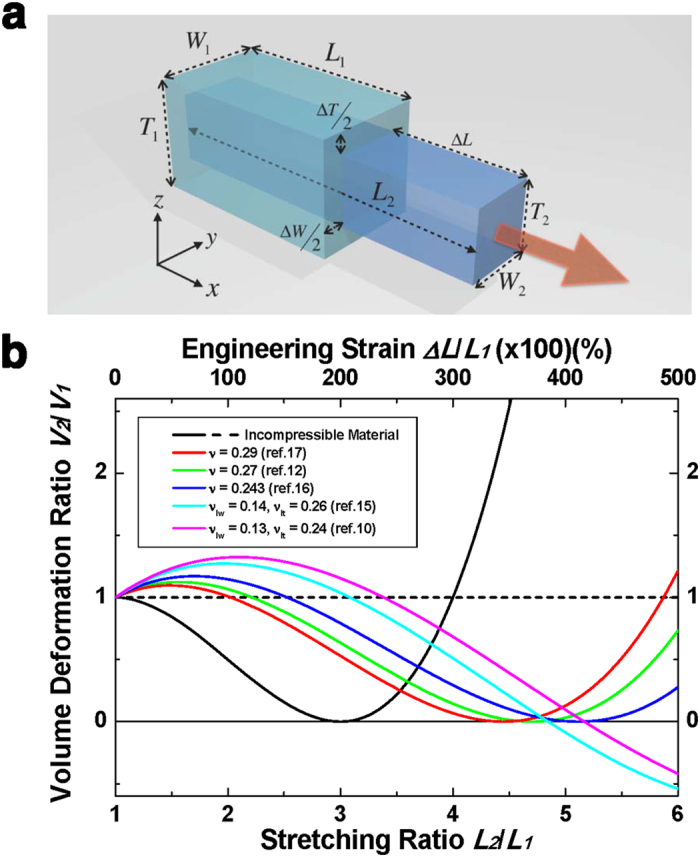
Volume deformations under tensile strain. (**a**) Schematic illustration of volume deformation in composite materials. Width and thickness decrease when length is elongated along the direction indicated by arrow. (**b**) Behavior of volume deformations under tensile strain for incompressible material and several composite materials from previous researches. Solid lines and dashed line (incompressible material only) show the calculated results based on linear elasticity (constant Poisson’s ratio) and nonlinear elasticity (strain-dependent Poisson’s ratio), respectively.

In previous researches, total volume deformation (*V*_*2*_) depending on applied strain has been calculated using the following equation that is built based on a large deformation assumption. For convenience of calculation, a six-sided structure was chosen as an example, as shown in Fig. 1a.


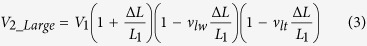


where *v*_*lw*_ and *v*_*lt*_ are Poisson’s ratios of the conductive composite in the direction of width and thickness (y and z axis in Fig. 1a); *L*_1_ and Δ*L* are the initial length of the material and the change in length with the strain, respectively. It is noted that *v*_*lw*_ and *v*_*lt*_ are same when the materials show isotropic property while they are different to each other for materials with anisotropic property. Details of the derivation process of equation (3) are demonstrated in Supplementary Note 1.

For comparison, the volume deformation with the applied tensile strain were calculated with constant Poisson’s ratio based on linear elasticity, by using previously reported Poisson’s ratio of the composite materials^10^,^12^,^15^,^16^,^17^ (solid lines in Fig. 1b). As the tensile strain increases, impractical behavior is predicted as shown in Fig. 1b. For the reported values of 0.243^16^, 0.27^12^, and 0.29^17^, the volume rapidly decreases with tensile strain around 70% and greater range and even becomes zero at larger tensile strain, as shown in Fig. 1b. This non-physical phenomenon come from using constant value of Poisson’s ratio under large deformation. According to Equation (3), the width and thickness of the deformed materials can become zero or negative number with constant Poisson’s ratio and certain value of tensile strain. In previous researches, they used constant Poisson’s ratio to calculated volume deformation under large deformation with Equation (3), showing limitations in analysis range. However, those erroneous calculations can be corrected when we consider strain-dependent Poisson’s ratio with nonlinear elasticity. The volume deformation of incompressible material was calculated to show the effect of the strain dependent Poisson’s ratio. It is noted that incompressible materials such as pure PDMS are typically considered to have a constant Poisson’s ratio of 0.5 at small strain. Although this value explains incompressible property at small strain, predicted volume decreases with tensile strain and vanishes at 200% engineering strain. The linear elasticity condition works quite well for predicting behavior of materials at low tensile strain. However, almost all stretchable conductive composite materials show high yield point exceeding 100% (L_2_/L_1_ = 2), while some exceptional materials can even reach up to 1000% (L_2_/L_1_ = 11)^15^,^24^,^25^. Therefore, all analytic conditions including Poisson’s ratio should satisfy large deformation assumption when we consider highly stretchable conductive composite materials. Poisson’s ratio of incompressible isotropic material can be expressed as below Equation (4) in engineering strain. Details of the procedures for calculation of Equation (4) are described in Supplementary Note 2.


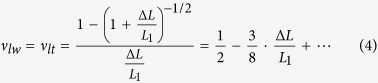


Equation (4) converges to 0.5 when ΔL is negligible, in other words, when small deformation assumption or linear elasticity condition is valid. However, in general conditions, we need to use Equation (4) as a strain-dependent Poisson’s ratio of incompressible isotropic material with nonlinear elasticity. The corresponding volume deformation of incompressible material under the tensile strain satisfying nonlinear condition are calculated with Equations (3) and (4) (dashed line in Fig. 1). The result shows consistent and physically reasonable data over entire strain range. We believe that analysis of the previous researches can be also improved if they consider strain dependency of Poisson’s ratio in large deformation with nonlinear elasticity. Therefore, for highly stretchable conductive composite materials, three-dimensional percolation theory embracing strain-dependent Poisson’s ratio should be used so that we can accurately analyze the whole dynamic range of the materials.

### Importance of accurate Poisson’s ratio and proper strain tensor

Poisson’s ratio is defined as the ratio of dimensional change in a specific direction with respect to the dimensional change in the direction that tensile strain is applied. As shown in Fig. 1a, Poisson’s ratios in width (*v*_*lw*_) and thickness (*v*_*lt*_) directions play important roles to determine the total volume deformation when tensile strain is applied in the length direction. Therefore, determination of accurate Poisson’s ratio of the composite materials is also important in the three-dimensional percolation theory because a small difference of Poisson’s ratio can result in a significant error in estimating the deformed total volume after stretching. It is well known that selection of measurement methods and strain tensor can greatly affect the measurement result of the elastic soft materials^20^,^21^,^22^. Nevertheless, few papers have dealt with this issue while reporting analysis results of the highly stretchable conductive composite materials using the three-dimensional percolation theory. As far as we know, constant Poisson’s ratios were used in previous researches without mentioning in detail how they determined the values. A video-type extensometer method and Cauchy strain tensor (also known as an engineering strain) have been widely used without further analysis^10^,^12^,^15^,^16^,^17^. Since Cauchy strain tensor is not suitable for a video-type measurement of large deformation case^20^, this method can generate a large error at certain tensile strain conditions. A contact-probe-type method can also be used instead of the video-type extensometer methods. However, they are reported to be inadequate in measuring properties of soft materials because of unreliability originated from unwanted deformations of specimen from the contact of the prove-tip and lack of full-field availability^20^.

**Figure 2:**
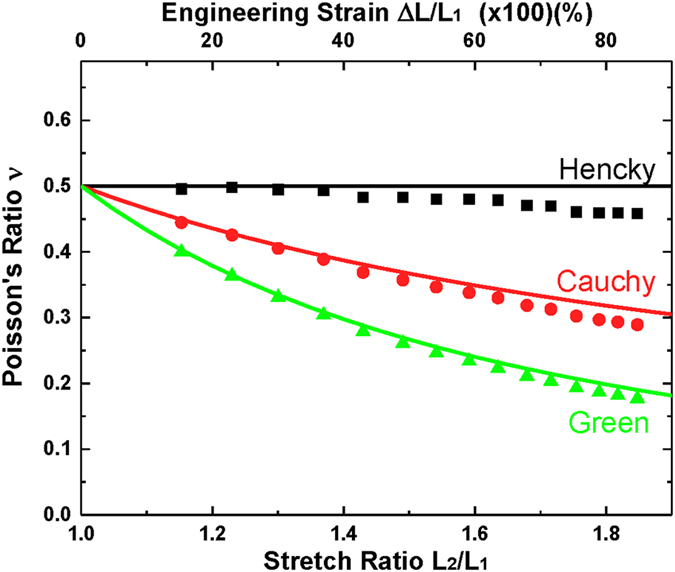
Poisson’s ratio of pure PDMS depending on strain definitions. Symbols and lines indicate experimentally measured results with the DIC method and calculated results (Supplementary Information, Note 2), respectively, using Hencky, Cauchy, and Green strain definitions.

In this research, we propose a non-contact, video-type method based on digital image correlation (DIC) to measure the dimensional change of the materials under several tensile strain conditions. Corresponding Poisson’s ratios are obtained by Hencky strain tensor and by using DIC software, VIC-2D (from correlated SOLUTIONS). In order to ensure validity of the DIC method and the Hencky strain tensor, a pure PDMS (SYLGARD 184, from Dow Corning) specimen was examined because it can be considered as an incompressible material. In fact, many reports have indicated that the Poisson’s ratio of pure PDMS is very close to 0.5 20,26. Detailed specimen fabrication and characterization methods are shown in the methods section and visualized results is shown in supplementary Fig. S1. Figure 2 shows Poisson’s ratios of a pure PDMS sample obtained from three strain tensors (Cauchy, Green, and Hencky) using the same DIC measurement. Results corresponding to Cauchy and Green strains are acquired by conversion of the measurement result based on Hencky strain tensor. It is noted that Hencky strain tensor showed Poisson’s ratio very close to 0.5 with experimental error of less than 10% over all strain ranges used for this experiment. The Cauchy and Green strain tensors, however, showed high strain dependency. Poisson’s ratios obtained from the Cauchy and Green strain definitions were 0.44 and 0.39 respectively, when the applied engineering strain was 15%. These values continuously decrease with the strain, then become 0.31 and 0.20 when the engineering strain was about 70%. Experimental results based on three different strains were verified by comparing with arithmetically calculated theoretical Poisson’s ratios of incompressible material that are shown as solid lines in Fig. 2. Details of the three tensors and procedures for Poisson’s ratio calculation are described in Supplementary Note 2. Since both experimental and calculated results are consistent with each other for all three cases, it is confirmed that the DIC method produces physically accurate measurement of dimensional change.

### Poisson’s ratios of isotropic and anisotropic composite materials

**Figure 3:**
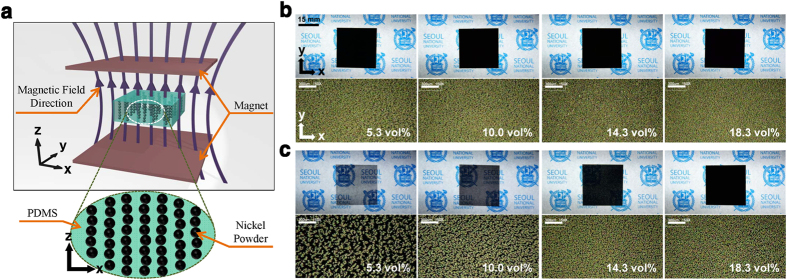
(**a**) Schematic illustration of nickel filler alignment with magnetic field and surface images of freestanding (**b**) ICCs and (**c**) ACCs. Each image set shows variation of (top) degree of light penetration and (bottom) filler structure, depending on alignment of nickel filler and concentration. All composites have thickness of 500 μm. (This figure is not covered by the CC BY license. The trademark of Seoul National University was used with permission from Seoul National University R&DB foundation. All rights reserved, used with permission).

In addition to the pure PDMS material, we also measured Poisson’s ratios for both isotropic and anisotropic composite materials (having isotropic and anisotropic Poisson’s ratios, respectively) by using the same DIC method with the Hencky strain tensor. All measured results were converted into values corresponding to engineering strain. We fabricated two-types of the composite materials consisting of PDMS and micro-sized spherical-shaped nickel powder (~5 μm diameter, from Sigma Aldrich) as matrix and conductive filler, respectively. One contains randomly distributed nickel filler; thus, it has an isotropic Poisson’s ratio and we call this “Isotropically distributed Conductive Composites” (ICC). As previously reported^27^, due to ferromagnetic behavior of the nickel powder, when magnetic field is applied to the composite material during curing process, the powder is aligned along the field direction, and thus, it has anisotropic Poisson’s ratio and will be called “Anisotropically aligned Conductive Composites” (ACC). Due to this nickel particle alignment, the ACC showed a negative-strain-dependency of the electrical performance while ICC showed a typical positive-strain-dependency when they are made into the stretchable electrodes^27^. The magnetic field was applied in thickness direction (z-axis) as shown in Fig. 3a. We define the direction of tensile strain as x-axis. Effect of the nickel filler alignment can also be visually verified even with the naked eye (Fig. 3b,c). Although these two composites have the same filler volume ratio, we can only see penetration of light through the ACC. This phenomenon is the result of alignment of nickel filler by magnetic field. In order to obtain Poisson’s ratios for both ICCs and ACCs, we examined all specimens using the DIC method applied on two surfaces of xy-plane (width) and xz-plane (thickness). Detailed fabrication and characterization methods are shown in the methods section.

**Figure 4:**
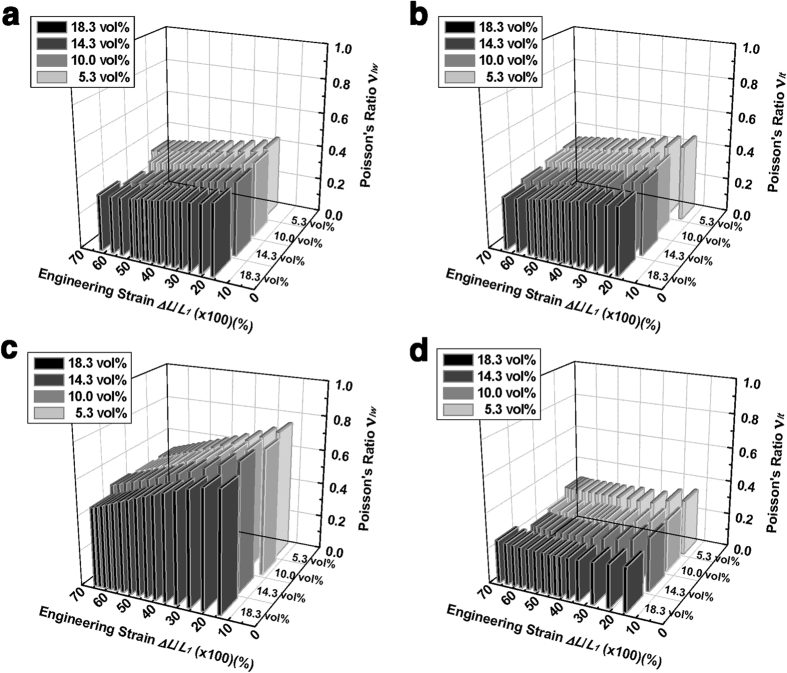
Poisson’s ratios depending on nickel filler content and strain with the DIC method. (**a**,**b**) Results of ICCs with (**a**) xy-plane(*v*_*lw*_) and (**b**) xz-plane(*v*_*lt*_). (**c**,**d**) Results of ACCs with (**c**) xy-plane(*v*_*lw*_) and (**d**) xz-plane(*v*_*lt*_).

Poisson’s ratios of ICCs and ACCs in terms of tensile strain for various filler concentrations (5.3 ~ 18.3 vol%) were measured as shown in Fig. 4. For ICCs, there were no significant differences between the Poisson’s ratios in width (*v*_*lw*_) (Fig. 4a) and thickness (*v*_*lt*_) (Fig. 4b) directions, which was measured from xy- and xz-plane DIC analyses, respectively. Although Poisson’s ratios of ICCs showed slight downward trend with the filler ratio increase, there were no significant variations. For ACCs, however, large anisotropy of Poisson’s ratios were observed for all samples. At low strain region, *v*_*lw*_ had very high values greater than 0.70 (Fig. 4c), and *v*_*lt*_ values were around 0.30 (Fig. 4d). When tensile strain increases, *v*_*lw*_ shows higher strain dependency than those values of ICCs, while *v*_*lt*_ remains constant around 0.30. Detailed results of Poisson’s ratios including measurement error are shown in Supplementary Fig. S2.

### Proposed 3-D percolation theory and comparison

Based on the Poisson’s ratio as a function of the applied tensile strain and the large deformation assumption the classical theory (Equation (1)) can be modified to the following three-dimensional percolation theory.


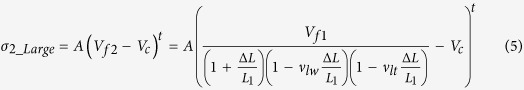


First, percolation threshold *V*_*c*_ and fitting exponent *t* can be determined by a least-square analysis of DC-conductivity^28^ (Supplementary Fig. S3) assuming *V*_*c*_ is constant as in the previously reported results. Best fit results indicated that percolation thresholds are 0.073 and 0.042 with fitting exponent of 6.321 ± 0.225 and 6.391 ± 0.052 for ICC and ACC, respectively. Although the same nickel particles were used for both ICC and ACC, the percolation threshold of ACC is lower than that of ICC. This can be explained by previous researches regarding increase in percolation network efficiency in aggregated filler structures^29^,^30^. It seems obvious that nickel particles in ACCs were aggregated by the applied magnetic field and that resulted in lower percolation threshold. ICC’s fitted threshold result of 0.073 corresponded well to the predicted value using the IPD approach with a concept of hardcore volume fraction^13^,^29^. Predicted result was 0.074, when the IPD approach was applied with 5 μm of filler particle diameter and 1.3 μm of interparticle distance. Scanning Electron Microscopy (SEM) image (Supplementary Fig. S4) showed that diameter of the nickel particle used in our experiment is nearly 5 μm.

Although *V*_*c*_ can be determined to be constant without considering deformation, it may not be true under the large deformation assumption. If there is directional anisotropy in the Poisson’s ratios or reorganization of composite due to the deformation, strain-dependent percolation threshold must be appropriately adjusted. Several researches have mentioned possibility of these effects^31^,^32^,^33^ and verified them with the experimental results^15^,^27^,^34^. In previous research^15^, the strain-dependent percolation threshold was only described by tensile strain. To more properly explain these effects, the percolation threshold must show dependency on Poisson’s effect as well as applied strain because volume change can directly affect variation of filler ratio or filler reorganization^35^. In addition, it is reasonable that strain-dependent percolation threshold have an exponential form, not a simplified linear form using a series expansion, for the infinite system and large deformation assumption^36^. Therefore, we introduced *V*_*c*_ as a function of the applied tensile strain and Poisson’s ratio as shown below.


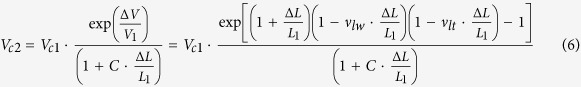



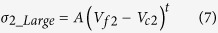


where *C* is a compensated constant. The compensated constant *C* can be explained as a weight factor of the effect of strain dependent percolation threshold. If the absolute value of *C* becomes larger, the degree of percolation threshold shift also becomes larger. We assume that the compensated constant *C* is correlated with types of materials used for stretchable matrix and conductive filler, and degree of anisotropic property.

**Figure 5:**
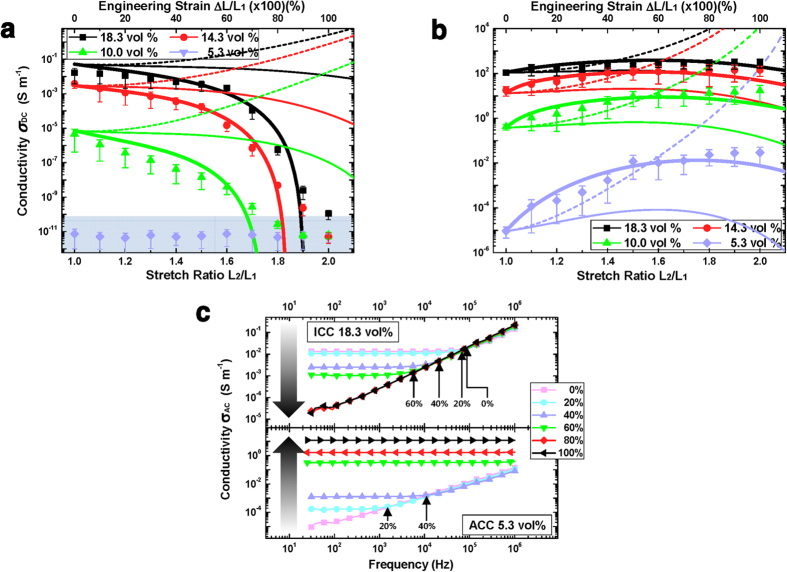
(**a**,**b**) Predicted and measured DC-conductivity in terms of tensile strain for (**a**) ICCs and (**b**) ACCs. Symbols indicate measured results. Dashed lines indicate the results of typical three-dimensional percolation theory (Equation (5) with constant Poisson’s ratio and constant percolation threshold). Thin solid lines indicate the results of modified three-dimensional percolation theory (Equation (5) with strain-dependent Poisson’s ratio and constant percolation threshold). Thick solid lines indicate the results of proposed three-dimensional percolation theory (Equation (7) with strain-dependent Poisson’s ratio and strain-dependent percolation threshold from equation (6)). (**c**) AC-conductivity in terms of frequency and tensile strain for (top) ICC (18.3 vol%) and (bottom) ACC (5.3 vol%). Arrows indicate the direction of strain increase.

The predicted results of the three three-dimensional percolation theories are plotted in Fig. 5 with experimental results of ICCs (Fig. 5a) and ACCs (Fig. 5b). The three theories are typical (Equation (5) with constant Poisson’s ratio and constant percolation threshold; dashed lines in Fig. 5), modified (Equation (5) with strain-dependent Poisson’s ratio and constant percolation threshold; thin solid lines in Fig. 5), and proposed (Equation (7) with strain-dependent Poisson’s ratio and strain-dependent percolation threshold from Equation (6); thick solid lines in Fig. 5) three-dimensional percolation theories. It is noted that, among three theories, the typical one is the same theory used in the previous researches. In our calculation, the constant Poisson’s ratio value was selected to be the value measured at near zero external tensile strain. It is noted that the measured result of the 5.3 vol% of ICC showed an insulating characteristic (measured as a noise level; shaded region in Fig. 5a) regardless of the applied tensile strain while the 5.3 vol% of ACC showed a somewhat conductive property (conductivity above 10^−5 ^S m^−1^) due to the magnetically aligned nickel particles^27^. When the fabricated ICCs and ACCs are analyzed with typical and modified theories, all results showed nearly the same characteristics up to 10% strain and were consistent with the measured results. However, as the strain becomes larger, the theoretical calculation results significantly differ from and show inconsistencies with the measured results. Under large strain, the predicted values with typical theory showed unreasonable prediction of unlimited increase in conductivity as the strain increases. The modified percolation theory including strain-dependent Poisson’s ratio showed consistent results over all strain ranges, although there were still somewhat differences from the measured results as the strain increases.

However, the proposed theory with the strain-dependent percolation threshold (Equation 6) showed good agreement with the measured results under all experimental conditions. The compensated constant *C* was obtained by fitting to the experimental results. In the case of ICCs, the compensated constants *C* were −0.23, −0.34 and −0.55 at 10.0, 14.3 and 18.3 vol%, respectively. The compensated constants *C* of ACCs were 1, 5, 7 and 3 at 5.3, 10.0, 14.3 and 18.3 vol%, respectively. Generally, the absolute values of the compensated constants showed an increasing tendency as the filler volume fractions increase. Therefore, in the case of ICCs, the strain-dependent percolation threshold *V*_*c*_(*ε*_*xx*_) showed decreasing tendency with the strain while, in the case of ACCs, the calculated results showed increasing tendency. The compensated predicted results by the proposed three-dimensional percolation theory showed obviously improved consistency with all measured results. The rapid decrease of ICC’s electrical conductivity at the region above 60% strain as well as ACC’s conductivity increase with the strain are also well explained.

We further analyzed our composite materials by using alternative current (AC) impedance spectroscopy to confirm the strain-dependent percolation threshold for both ICCs and ACCs. The impedance spectroscopy is well known to be utilized to monitor the formation of conductive paths of the conductive composite materials^37^,^38^,^39^. Previous researches showed that, during AC impedance measurement, when the measured conductivity becomes independent of frequency, the conductive paths have started to form in the composite materials. We performed 2-terminal AC impedance spectroscopy using an Agilent 4284a LCR meter. For all samples, several tensile strains were applied during the AC impedance spectroscopy measurement. Detailed characterization methods are shown in the methods section. The most noticeable results of two conditions are shown in Fig. 5c (other results are shown in Supplementary Fig. S5). In the case of ICCs, increase of percolation threshold with the strain was most noticeable for the 18.3 vol% composite. Initially, it shows a frequency-independent conductivity over certain frequency range but as strain increases the frequency-independent range shrinks indicating percolation threshold change with the strain, and at high strain (>60%), it does not show the frequency-independent conductivity over all frequency range. This means that the ICC loses DC-conductivity at large strain conditions. In the case of ACCs, on the contrary, decrease of percolation threshold with strain was noticeable due to its negatively strain-dependent resistance^27^. Since, at all concentrations other than 5.3 vol%, ACCs showed good DC-conductivity (thus the percolation paths are already made), only the low concentration ACC showed an obvious percolation threshold change. However, all ACCs showed increasing conductivity with the strain as previously reported^27^. We assume that the strain-dependent percolation threshold could be improved even though this phenomenon has agreed well with our experimental results and was verified by the impedance spectroscopy. Other parameters can also be applied to the strain-dependent percolation threshold such as filler particle shape, mechanical properties of elastomer matrix and complex deformation conditions. Additional research on the strain-dependent percolation threshold may yield interesting results for various composite systems.

To conclude, we revisited the classical three-dimensional percolation theory and proposed an improved theory for accurate analysis of highly stretchable conductive composite materials. The proposed theory was based on nonlinear elasticity including the strain-dependent Poisson’s ratio and the strain-dependent percolation threshold. In addition, precise determination of Poisson’s ratio using the DIC method with the Hencky strain tensor was introduced. We were able to accurately predict volume deformation of the highly stretchable conductive composites and thus the behavior of the composite materials with both isotropic and anisotropic Poisson’s ratios. Although our approach does not include the entire range of loading conditions and deformation types, we believe that the proposed theory and method can be used as a stepping stone for further analysis of various highly stretchable conductive composite materials under complex environment.

## Methods

### Fabrication Methods

(*The pure PDMS specimen*) 10:1 weight ratio mixture of PDMS (Sylgard 184 from Dow Corning) base and curing agent was mixed for 10 minute and then put in a desiccator for 1 hour to degas air in it. The degassed mixture was poured onto a flat aluminum mold and then cured in a convection oven at 130 °C for 30 min. The cured pure PDMS was cut in the shape of straight-bar with 50-mm length, 3-mm width and 3-mm thickness for DIC and electrical measurements.

(*Isotropically distributed Conductive Composites (ICCs*)) 10:1 weight ratio mixture of PDMS was mixed with various volume ratio of nickel powder (average particle size ~5 μm, 99.99% from Sigma Aldrich). To make ICCs, the mixtures of PDMS and nickel powder were directly cured in the convection oven at 130 °C for 30 min. The cured ICCs were cut into the same size as the pure PDMS specimen.

(*Anisotropically aligned Conductive Composites (ACCs*)) To make ACCs, the aluminum mold filled with the mixture of PDMS and nickel powder was placed between two neodymium magnets and left in room temperature for 10 minute for the nickel filler alignment. Finally, it was cured in the convection oven at 130 °C for 30 min, and cut in the same shape as the bare PDMS specimen.

### Characterizations

(*Digital Image Correlation (DIC*)) All specimens had 50-mm length, 3-mm width and 3-mm thickness. In order to apply a speckle pattern onto those surfaces, the pure PDMS specimen was spray painted with black colored matt-type spray. In contrast, ICCs and ACCs were spray painted with white colored matt-type spray because they have black colored surfaces. The average diameter of speckles was 50 μm. Then 40-times magnified surface images were obtained, with a uni-axially stretched condition along length (x-axis) under 0 to 100% tensile strain. Images had a resolution of 640 × 480 pixels. In order to consider the mechanical anisotropy of ACCs, we examined ICCs and ACCs with the DIC method at two surfaces of xz-plane (thickness) and xy-plane (width). Axial strain and transverse strain were acquired by comparing initial and deformed state by using DIC software, VIC-2D (from correlated SOLUTIONS). Parameters of VIC-2D were subset size of 45 pixels with three steps and decay filter was used with filter size of 15. Finally Poisson’s ratio was calculated from the measured axial and transverse strains.

(*Impedance Spectroscopy*) 2-terminal AC impedance spectroscopy was performed using an Agilent 4284a LCR meter with DC potential 5 V and voltage amplitude 0.1 V. The frequency range was from 20 to 10^6 ^Hz. All Specimens were measured under strain of 0%, 20%, 40%, 60%, 80% and 100%.

(*DC Conductivity*) DC-conductivity was calculated from measured DC-resistance. DC-resistance was measured using two digital electrometers when specimens were being stretched. Keithley 6517B was used for range between 200 TΩ and 200 MΩ, and Keithley 2400 was used for range below 200 MΩ.

(*Stretching Condition*) In all experiments including DIC, impedance spectroscopy, and DC conductivity, tensile loading was applied with homemade stretching equipment with an 83.3 μm/s of constant stretching speed at a room temperature of 20 °C.

## Additional Information

**How to cite this article**: Kim, S. *et al.* Revisit to three-dimensional percolation theory: Accurate analysis for highly stretchable conductive composite materials. *Sci. Rep.* **6**, 34632; doi: 10.1038/srep34632 (2016).

## Supplementary Material

Supplementary Information
