# Supplementary material for: Revisit to three-dimensional percolation theory: Accurate analysis for highly stretchable conductive composite materials
Source: Sci Rep. 2016 Oct 3;6:34632. doi: 10.1038/srep34632 (PMC5046142; doi:10.1038/srep34632)
Supplement: Supplementary Information [file srep34632-s1.pdf]

Revisit to three-dimensional percolation theory:

Accurate analysis for highly stretchable conductive composite materials

Supplementary Information

Sangwoo Kim, Seongdae Choi, Eunho Oh, Junghwan Byun, Hyunjong Kim, Byeongmoon Lee, Seunghwan Lee, and Yongtaek Hong*

Department of Electrical and Computer Engineering, Inter-University Semiconductor Research Center (ISRC), Seoul National University, Seoul 08826, Republic of Korea

*Corresponding Author (email:yongtaek@snu.ac.kr)

Supplementary Note 1: Deriving of volume deformation and 3-D percolation theory under elongated condition.

If a six-sided material is subjected to uniaxial tensile strain, the material will be elongated along the direction the tensile strain is applied, and shrink (Poisson's Ratio $\nu > 0$) or expand ($\nu < 0$) along perpendicular directions to the tensile strain according to the Poisson effect (Fig. 1a). Direction of volumetric change of this material can follow three different situations depending on its value of Poisson's ratio. Volume will increase ($-1<\nu<0.5$) or remain unchanged ($\nu = 0.5$), with applied uniaxial tensile strain in linear elasticity.

If the material is elongated along x-axis, length of three axes after deformation is given by:

$$L_2 = L_1 + \Delta L \quad \text{(S1)}$$

$$W_2 = W_1 - \Delta W \quad \text{(S2)}$$

$$T_2 = T_1 - \Delta T \quad \text{(S3)}$$

where L_1, W_1, T_1 and L_2, W_2, T_2 are the length, width, thickness of the material before and after tensile strain is applied, respectively. ΔL, ΔW, ΔT is the change in length, width, thickness.

The definition of the Poisson's ratio is given by:

$$1 - \nu_{lw}\frac{\Delta L}{L_1} = 1 - \frac{\Delta W}{W_1} \quad \text{(S4)} \quad \text{and} \quad 1 - \nu_{lt}\frac{\Delta L}{L_1} = 1 - \frac{\Delta T}{T_1} \quad \text{(S5)}$$

where ν_{lw} and ν_{lt} are Poisson's ratios of the conductive composite in the direction of the width and thickness and ε_{xx} is the tensile strain along the direction of the length.

Then, a deformed volume under small deformation condition is given as below.

$$V_{2\_Small} = V_1\left(1 + \frac{\Delta L}{L_1}\right)\left(1 - \nu_{lw}\frac{\Delta L}{L_1}\right)\left(1 - \nu_{lt}\frac{\Delta L}{L_1}\right) \quad \text{(S6)}$$

The classical percolation theory (Equation (1)) becomes the following two equations according to deformation conditions.

$$\sigma_{2\_Small} = A\left[\frac{V_f}{\left(1+\frac{\Delta L}{L_1}\right)\left(1-\nu_{lw}\frac{\Delta L}{L_1}\right)\left(1-\nu_{lt}\frac{\Delta L}{L_1}\right)} - V_c\right]^t \qquad \text{(S7)}$$

Supplementary Note 2: Details of tensors and procedure for Poisson's ratio calculation of incompressible Material[S1,S2].

Stretch ratio is defined as below when uniaxial tension along length direction is applied to materials.

$$\lambda_x = \frac{L_2}{L_1} \tag{S8}$$

Three strains, the Cauchy (ε_{xx}^C), Green (ε_{xx}^G) and Hencky (ε_{xx}^H), are defined as below.

$$\varepsilon_{xx}^C = \frac{L_2 - L_1}{L_1} = \lambda_x - 1 \tag{S9}$$

$$\varepsilon_{xx}^G = \frac{{L_2}^2 - {L_1}^2}{2{L_1}^2} = \frac{1}{2}\left({\lambda_x}^2 - 1\right) \tag{S10}$$

$$\varepsilon_{xx}^H = \ln\frac{L_2}{L_1} = \ln\lambda_x \tag{S11}$$

In the case of an incompressible isotropic material, relations among stretch ratios along three axes are as below.

$$\lambda_y = \lambda_z \tag{S12}$$

$$\lambda_x\lambda_y\lambda_z = 1 \tag{S13}$$

$$\lambda_y = {\lambda_x}^{-1/2} \tag{S14}$$

Poisson's ratios (y-direction) depending on each definition of strain tensor are calculated with below equations.

$$\nu_{xy}^C = -\frac{\varepsilon_y^C}{\varepsilon_x^C} = \frac{1-(1+\varepsilon_x^C)^{-1/2}}{\varepsilon_x^C} \approx \frac{1}{2} - \frac{3}{8}\varepsilon_x^C + \cdots \tag{S15}$$

$$\nu_{xy}^G = -\frac{\varepsilon_y^G}{\varepsilon_x^G} = \frac{1}{2(1+\varepsilon_x^C)(1+\varepsilon_x^C/2)} \approx \frac{1}{2} - \frac{3}{4}\varepsilon_x^C + \cdots \tag{S16}$$

$$\nu_{xy}^H = -\frac{\varepsilon_y^H}{\varepsilon_x^H} = -\frac{\ln\left(1+\varepsilon_y^C\right)}{\ln\left(1+\varepsilon_x^C\right)} = -\frac{\ln\lambda_y}{\ln\lambda_x} = -\frac{\ln\left({\lambda_x}^{-1/2}\right)}{\ln\lambda_x} = \frac{1}{2} \tag{S17}$$

Only the Hencky's ratio (equation S17) shows value of 0.5 without strain dependency while others show strain dependency when the applied tensile strain becomes larger.

S1. Starkova, O. & Aniskevich, A. Poisson's ratio and the incompressibility relation for various strain measures with the example of a silica-filled SBR rubber in uniaxial tension tests. *Polym. Test.* **29,** 310–318 (2010).

S2. McClung, A. J. W., Tandon, G. P., Goecke, K. E. & Baur, J. W. Non-contact technique for characterizing full-field surface deformation of shape memory polymers at elevated and room temperatures. *Polym. Test.* **30,** 140–149 (2011).

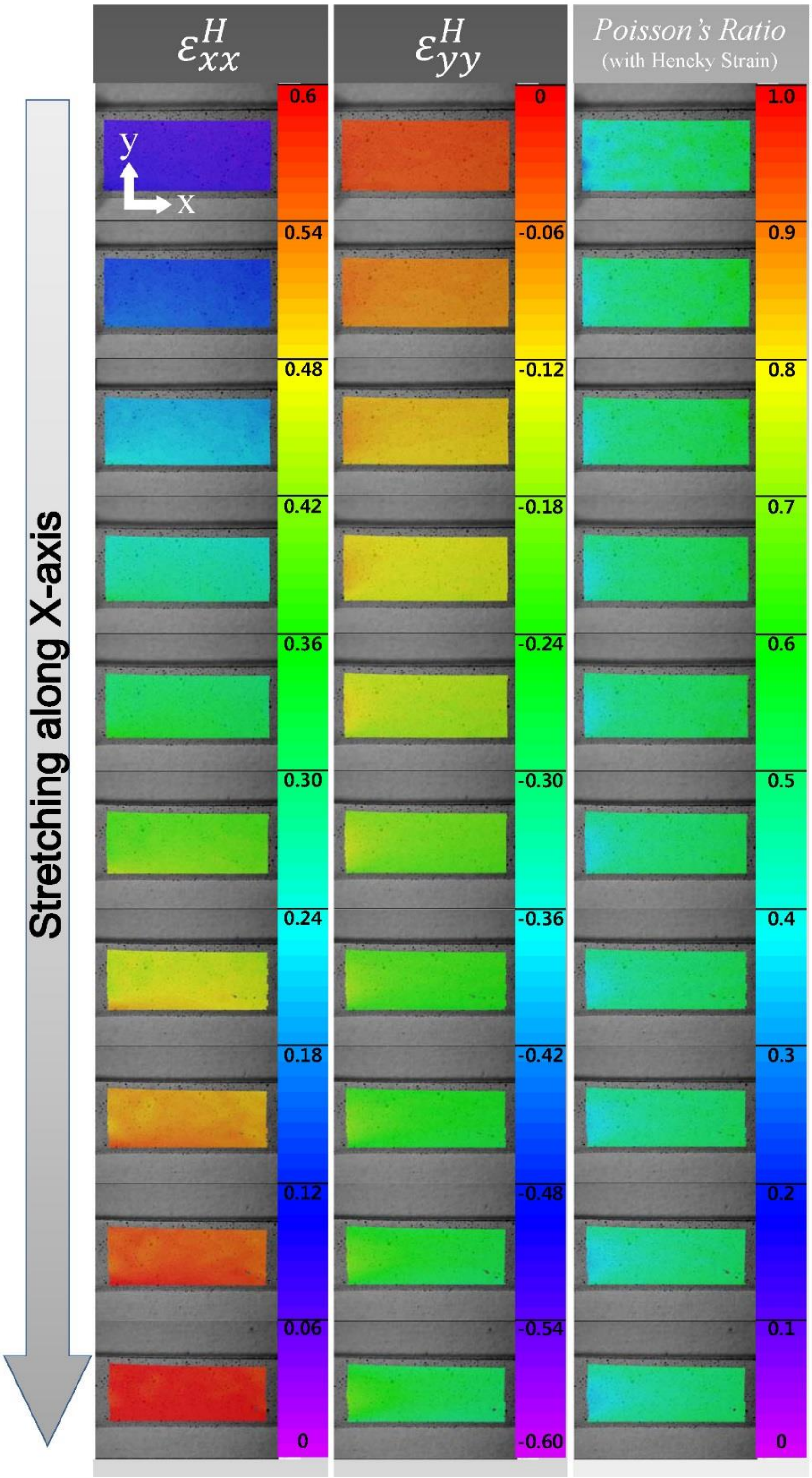

$avg.E_{xx}^{H}$ (%)	$avg.E_{yy}^{H}$ (%)	Poisson's Ratio
( ):equivalent values of Engineering strain		
0.0753172 (0.0782261)	-0.0347559 (-0.0341589)	0.463673 (0.436668)
0.142298 (0.152920)	-0.0704043 (-0.0679831)	0.495632 (0.444566)
0.20709 (0.230093)	-0.103041 (-0.0979100)	0.498173 (0.425523)
0.262696 (0.300431)	-0.12988 (-0.121800)	0.494788 (0.405417)
0.314377 (0.369406)	-0.15505 (-0.143631)	0.493435 (0.388816)
0.399002 (0.490337)	-0.192561 (-0.175156)	0.482715 (0.357216)
0.464592 (0.591365)	-0.222975 (-0.199865)	0.480053 (0.337973)
0.518497 (0.679501)	-0.243997 (-0.216510)	0.470633 (0.318631)
0.562357 (0.754804)	-0.258981 (-0.228162)	0.460554 (0.302280)
0.597659 (0.817858)	-0.274327 (-0.239917)	0.459018 (0.293347)

Figure S1 Result of DIC analysis for bare PDMS. (left) ε_{xx}^{H}, ε_{yy}^{H} and Poisson's ratio maps depicted by color gradation depending on 1-D stretching along x-axis and (right) average values of those results. Values in parentheses indicate converting results equivalent to engineering strain.

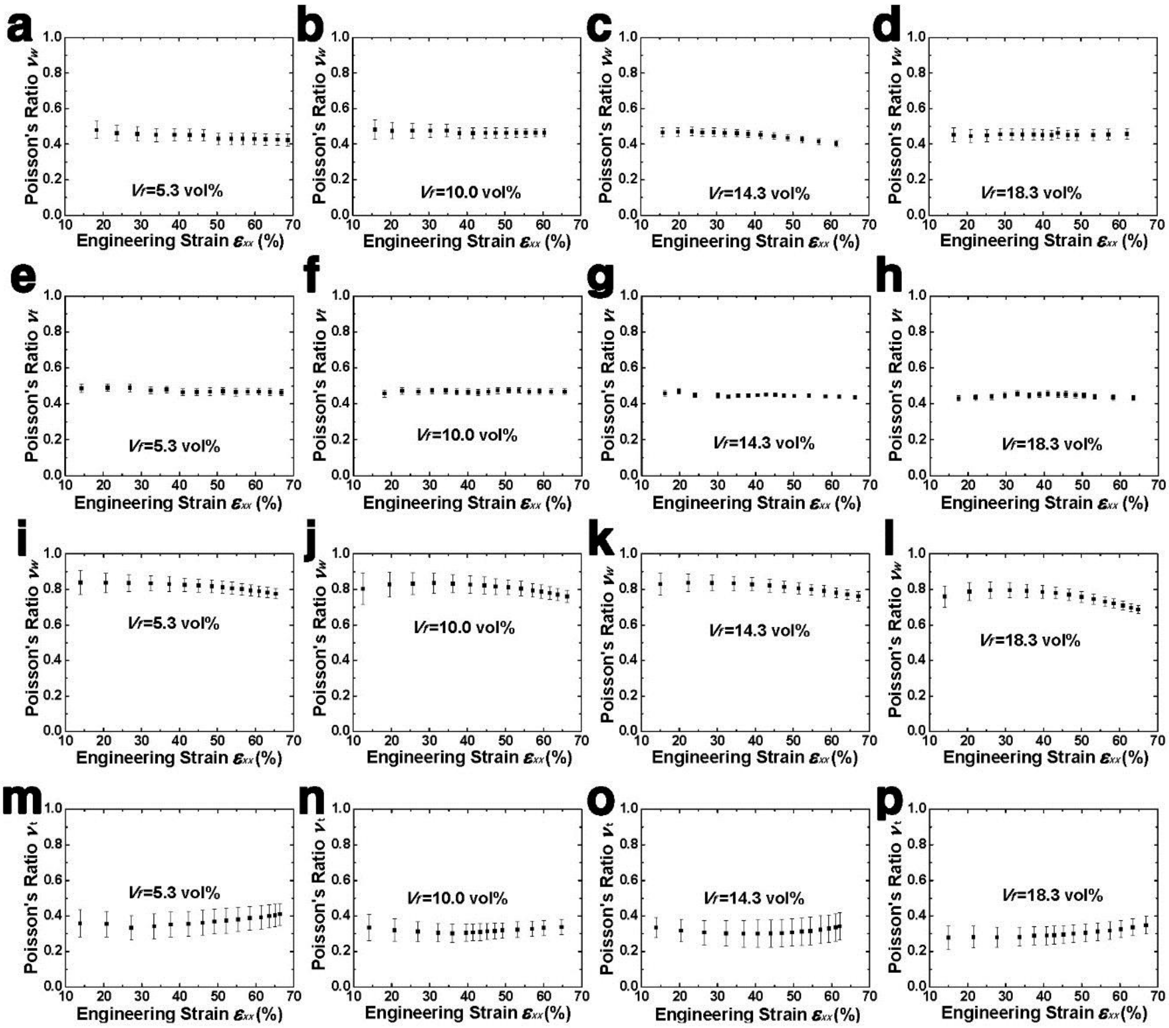

Figure S2 Measured Poisson's ratio for different nickel filler contents for (a-d) ICCs with width direction, (e-h) ICCs with thickness direction, (i-l) ACCs with width direction and (m-p) ACCs with thickness direction.

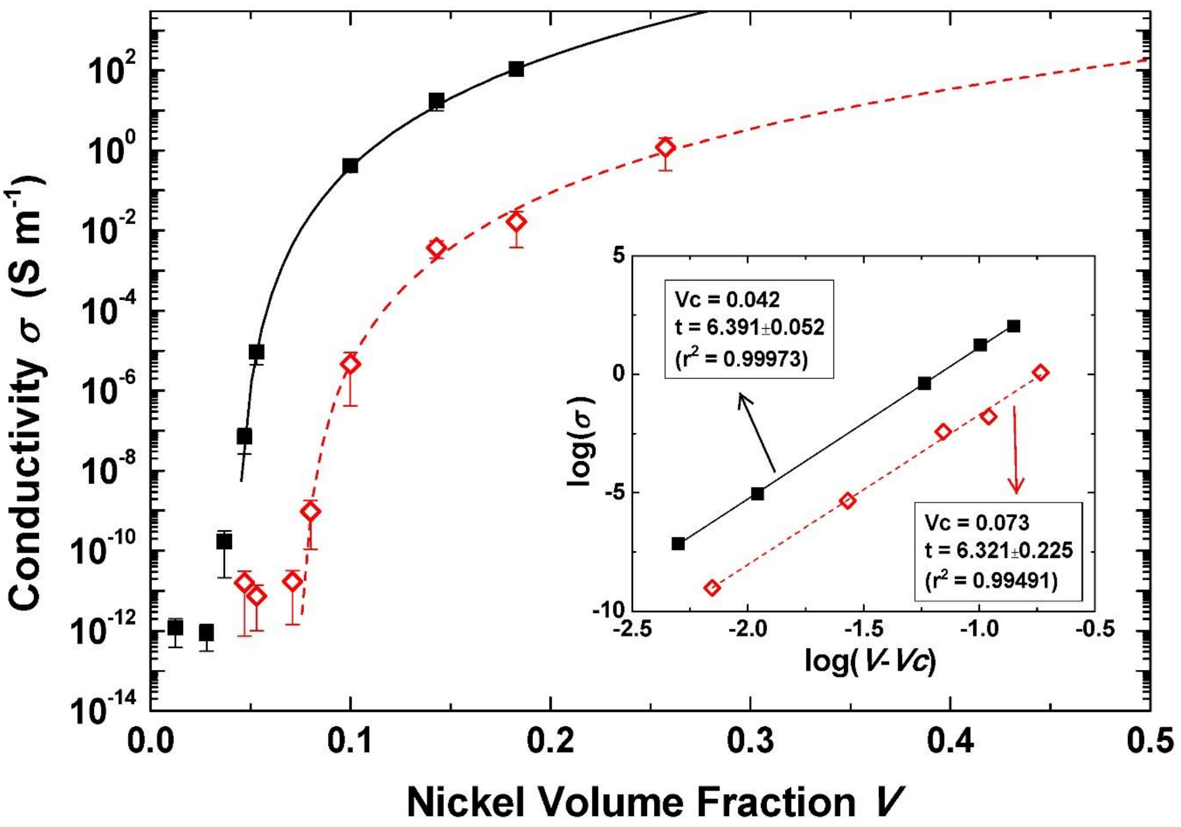

Figure S3 DC-conductivity of ACCs (black filled boxes) and ICCs (red hollow diamonds) in terms of nickel volume fraction. The inset shows a log-log plot of the DC-conductivity as a function of V_f - V_c. Black solid lines and red dashed lines correspond to the least square fitting of the experimental results of ACCs and ICCs respectively.

Supplementary Note 3: Interparticle Distance Model

$$V_{c\_sphere} = \frac{\pi D^3}{6(D + D_{IP})^3} \quad \text{(S18)}$$

where, V_{c_sphere} is the percolation threshold of random distributed composite system using spherical type filler particle. D and D_{IP} are the diameter of the filler particle and the interparticle distance, respectively[S3].

The diameter of the filler particle was determined as 5 μm, based on the cross-sectional SEM (Scanning Electron Microscope) image (Figure S3). And the interparticle distance was chosen as 1.3 μm.

This condition gives, $V_{c\_Overall}$ = 0.261 and $V_{c\_Hardcore}$ = 0.074.

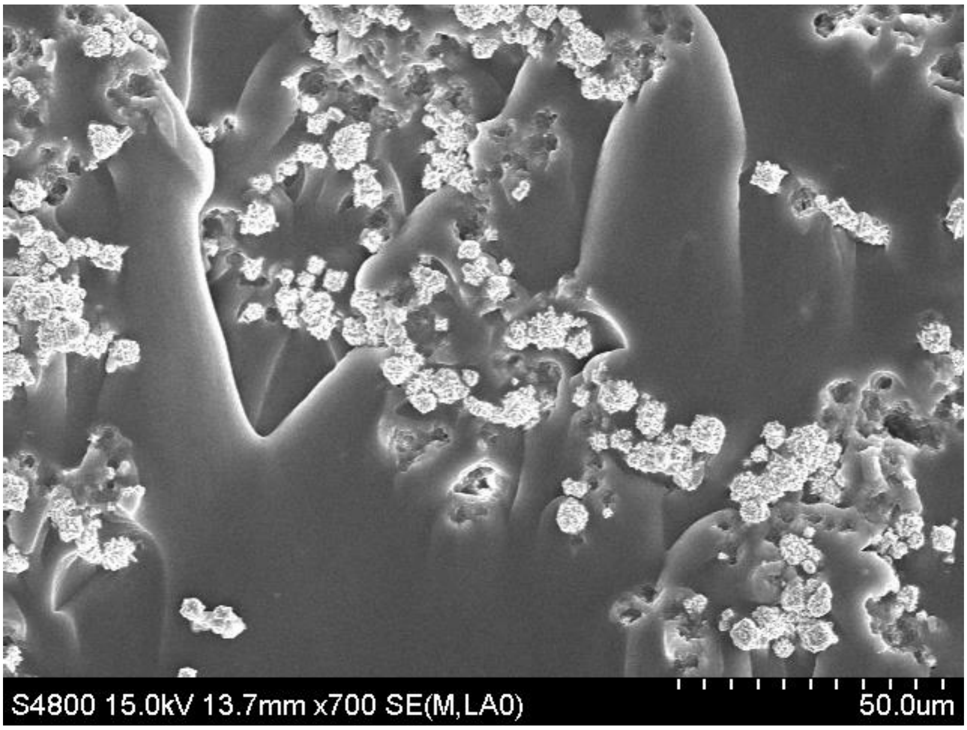

Figure S4 SEM image of nickel particles in PDMS matrix.

S3. Li, J. & Kim, J.-K. Percolation threshold of conducting polymer composites containing 3D randomly distributed graphite nanoplatelets. *Composites Science and Technology* **67,** 2114–2120 (2007).

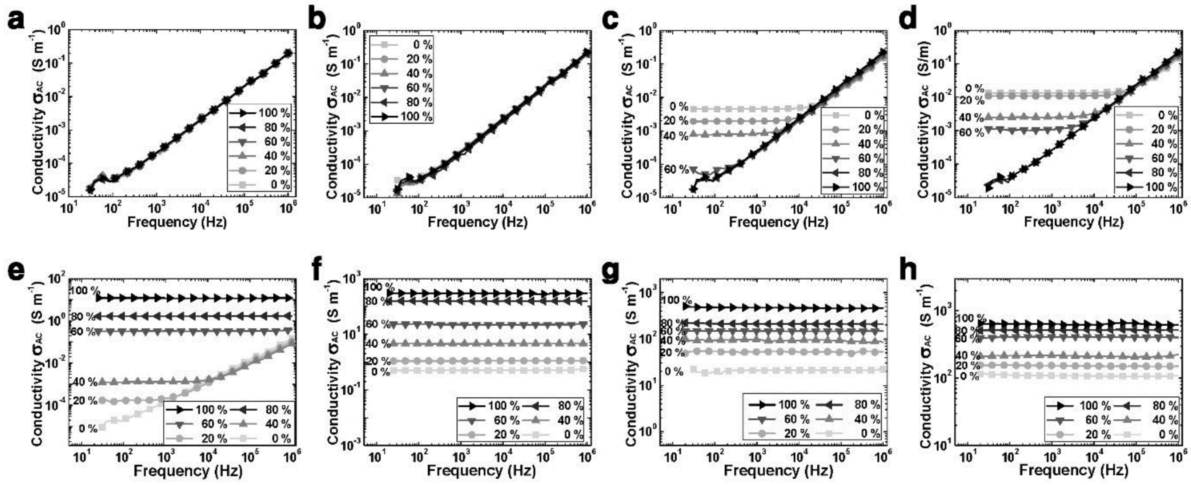

Figure S5 AC-conductivity in terms of frequency and tensile strain with different nickel filler content for (a-d) ICCs with 5.3, 10.0, 14.3, and 18.3vol% and (e-h) ACCs with 5.3, 10.0, 14.3, and 18.3vol%
